# Rapid acquisition of polymorphic virulence markers during adaptation of highly pathogenic avian influenza H5N8 virus in the mouse

**DOI:** 10.1038/srep40667

**Published:** 2017-01-17

**Authors:** Won-Suk Choi, Yun Hee Baek, Jin Jung Kwon, Ju Hwan Jeong, Su-Jin Park, Young-il Kim, Sun-Woo Yoon, Jungwon Hwang, Myung Hee Kim, Chul-Joong Kim, Richard J. Webby, Young Ki Choi, Min-Suk Song

**Affiliations:** 1 College of Medicine and Medical Research Institute, Chungbuk National University, Cheongju, Republic of Korea; 2 Viral Infectious Disease Research Center, Korea Research Institute of Bioscience and Biotechnology, Daejeon 34141, South Korea; 3 Microbiomics and Immunity Research Center, Korea Research Institute of Bioscience and Biotechnology, Daejeon 34141, South Korea; 4 College of Veterinary Medicine, Chungnam National University, Dae Jeon 305-764, Republic of Korea; 5 Department of Infectious Diseases, St. Jude Children’s Research Hospital, Memphis, TN 38105, USA

## Abstract

Emergence of a highly pathogenic avian influenza (HPAI) H5N8 virus in Asia and its spread to Europe and North America has caused great concern for human health. Although the H5N8 virus has been only moderately pathogenic to mammalian hosts, virulence can still increase. We evaluated the pathogenic potential of several H5N8 strains via the mouse-adaptation method. Two H5N8 viruses were sequentially passaged in BALB/c mice and plaque-purified from lung samples. The viruses rapidly obtained high virulence (MLD_50_, up to 0.5 log10 PFU/mL) within 5 passages. Sequence analysis revealed the acquisition of several virulence markers, including the novel marker P708S in PB1 gene. Combinations of markers synergistically enhanced viral replication and polymerase activity in human cell lines and virulence and multiorgan dissemination in mice. These results suggest that H5N8 viruses can rapidly acquire virulence markers in mammalian hosts; thus, rapid spread as well as repeated viral introduction into the hosts may significantly increase the risk of human infection and elevate pandemic potential.

Since the highly pathogenic avian influenza (HPAI) H5N1 virus was first isolated in China in 1996^1^, these viruses have maintained stable serotypic combinations between the haemagglutinin (HA) and neuraminidase (NA) genes, though they are genetically divided into 10 distinct clades (0–9) based on the divergence of the HA gene^2^. Despite the antigenic divergence of the HPAI H5N1 viruses and their increasing infectiousness in humans, no one has found direct evidence of human-to-human transmission. However, a major antigenic shift in the H5N1 virus began in 2008 in China, with a highly pathogenic H5N5 virus^3^. A major, prolonged outbreak of HPAI H5 virus expressing the novel N8 gene began in South Korea in early 2014 and subsequently spread to Japan and Europe^4^. Moreover, the Eurasian HPAI H5N8 virus crossed from Eurasia to North America for the first time and created novel reassortants with North American viruses^4^,^5^. Rapid spread and reassortment events severely increase the risk of human infection and pandemic.

In general, avian influenza viruses (AIVs) have strong host-species barriers^6^. Nevertheless, recent human infections with various AIVs, including H5N1, H5N6, H7N3, H7N7, H7N9, H9N2, and H10N8^7^,^8^,^9^,^10^,^11^,^12^,^13^,^14^, pose substantial risks to public health and highlight the need to identify the viral factors that enable cross-species transmission. AIVs must acquire many molecular changes to overcome host-species barriers, such as changes in the receptor-binding region of HA and polymerase subunits, including PA, PB1, and PB2. In particular, adaptive mutations in the polymerase complex alter polymerase activity in mammalian cell lines, potentially enhancing viral growth efficiency and virulence. Substitution of glutamic acid (E) with lysine (K) at position 627 in the PB2 gene affects the polymerase’s activity and the AIVs’ replication efficiency in mammals^15^,^16^,^17^,^18^,^19^. In addition to the E627K mutation in the PB2 gene, D701N, Q591R/K, and T271A mutations are associated with increased viral growth and/or pathogenicity in mammalian cells/hosts^18^,^20^,^21^,^22^. Adaptive mutations, including T97I, K142E, and I353R, in avian-originated PA genes also contribute to enhanced polymerase activity and viral replication in mammalian cells and hosts^16^,^23^,^24^. Furthermore, a combination of the mutations synergistically enhances avian influenza virulence and replicative efficiency in mammalian hosts *in vitro* and *in vivo*^25^,^26^.

Although the molecular basis of avian influenza adaptation to mammalian species has been intensively studied, the diverse genetic characteristics of influenza virus support strain/lineage-specific factors associated with cross-species adaptation^27^,^28^, implying that we need to understand the potential adaptive factors, especially when the newly emerging virus is circulating and has pandemic potential. Therefore, we investigated whether the novel HPAI H5N8 virus, which exhibits modest virulence in mammalian hosts^29^,^30^, could acquire high pathogenicity in a mouse model. We also identified molecular changes associated with increased virulence.

## Results

### Rapid acquisition of the virulent phenotype of avian influenza H5N8 viruses in mice

**Figure 1:**
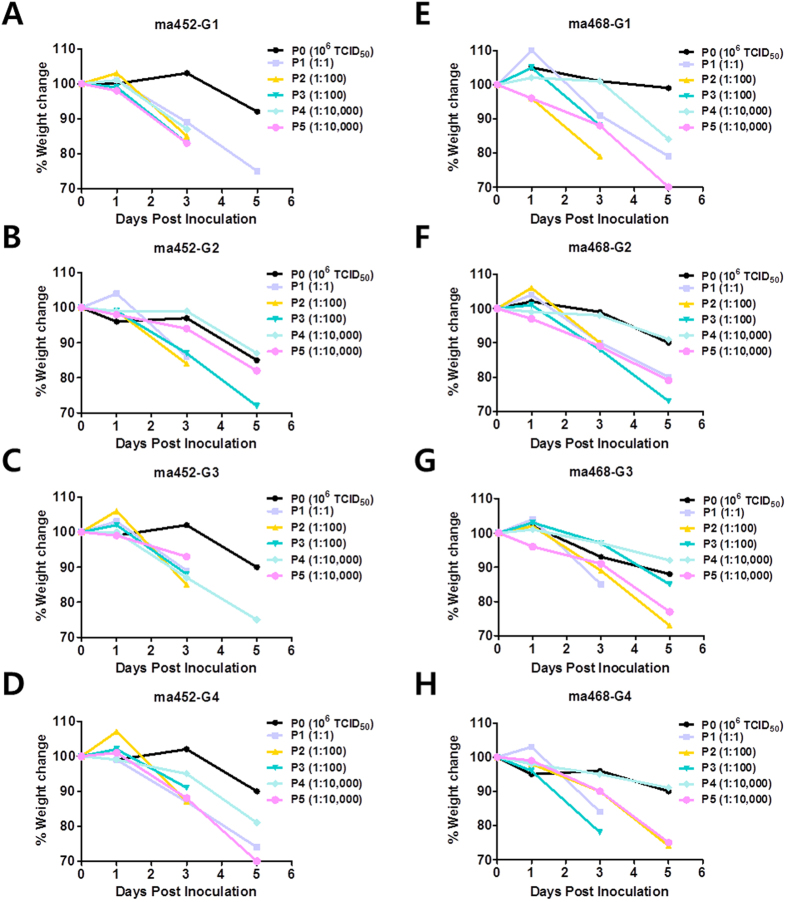
Weight changes in mice infected with H5N8 viruses during lung-to-lung sequential passages. Groups of 4 mice were infected with initial doses of 30 μl of 10^7 ^PFU/ml of either W452 (**A**–**D**) or W468 (**E**–**H**), and their body weights were monitored for 5 days. The lungs of infected mice were collected at 5 dpi, and the supernatants of lung homogenates were independently and sequentially passaged 5 times in mice. Individual passage numbers are indicated as P0-P5, with dilution factors.

To investigate whether HPAI H5N8 virus, which causes modest disease in mammalian hosts such as mice and ferrets^29^,^30^ can be increased in virulence, we performed sequential lung-to-lung passages in mice. Two H5N8 viruses, Mallard duck/Korea/W452/2014 (W452) and Environment/Korea/W468/2015 (W468), share common genetic characteristics (i.e., 98.9–99.7% homology; see Supplementary Table S1) but were isolated in 2 waves of an outbreak in 2014 and 2015, respectively, in South Korea^31^. For this experiment, we intranasally (i.n.) inoculated 4 mice with 30 μL of 10^7.0^ plaque-forming units (PFU)/mL of the viruses and independently passaged the viruses to verify their common genetic phenotypes after virulence arose. During passage 0 (P0), all of the infected mice showed moderate weight loss (1–15% of their initial body weight) until 5 days post inoculation (dpi) (Fig. 1). During P1, 50% of the infected mice were killed or euthanized because they lost more than 25% of their body weight within 5 dpi. Thus, we prepared a 1:100 dilution of the supernatant of the lung homogenate from P1 mice and inoculated P2 naïve mice. We observed that 100% of infected P2 mice died or lost more than 25% of their body weight at 5 dpi. Similar results were observed in P3 mice. Thus, we further diluted lung homogenate from P3 mice to 1:10,000 and inoculated P4 and subsequently P5 mice. Dramatically increased morbidity was still observed in P5 mice, even at a low-inoculation dosage (10,000-fold diluted from lung homogenate of P4 mice). These results indicate that H5N8 viruses can achieve virulence in mice within 5 passages.

### Molecular changes in the genomes of H5N8 viruses after lung-to-lung passage in mice

To investigate how molecular changes in mouse-adapted (ma) H5N8 viruses potentially contribute to increased virulence in mice, we performed whole-genome sequencing of 8 viruses obtained from the lung homogenates of the P5 mice. We found various mutations in the following viral genes: PA, PB1, PB2, HA, and nucleoprotein (NP). Interestingly, 2 mutations, D701N and E627K, were common but strain specific to the ma452 and ma468 viruses, respectively (see Supplementary Table S2). Some representatives of both maH5N8 viruses acquired common mutations: P708S in PB1, T97I in PA, and A156T in HA genes. Other mutations (Q591K in PB2; F4V, M86I, and Q556R in PA; K40E, S181T, L322P, V395I, and K475N in HA; and M448I and G485R in NP) were also found in some of maH5N8 viruses (i.e., ma452-G1, ma452-G2, ma468-G1, and ma468-G4). Single amino acid polymorphism analysis revealed that the rates of certain mutations (e.g., A156T in HA and E627K and Q591K in PB2) were increased in human isolates compared to avian isolates (see Supplementary Table S3).

### *In vitro* characterization of mouse-adapted and plaque-purified H5N8 viruses

To verify the molecular properties of the maH5N8 viruses, we plaque-purified them from the P2 to P5 lungs and then fully sequenced those viruses (Table 1). Most plaque-purified viruses carried the mutations found in their parental lung-passaged viruses. The plaque morphologies of the ma/purified H5N8 viruses varied from 0.3 to 2.15 mm in Madin-Darby canine kidney (MDCK) cells, and the well-known virulence markers E627K and D701N in PB2 were not associated with significantly different plaque size compared to that of plaques from wild-type viruses (Table 1 and see Supplementary Fig. S1). PB1 P708S (ma468 G2-1) was associated with dramatically increased plaque size in combination with PB2 E627K compared to wild-type W468 (0.34 *vs* 1.45, *p* < 0.05), and PA Q556R and/or HA K475N were also related to the increased plaque size of the ma452 G3-2 virus compared to W452 (0.43 *vs* 1.07, *p* < 0.05) (Table 1 and see Supplementary Fig. S1).

**Figure 2:**
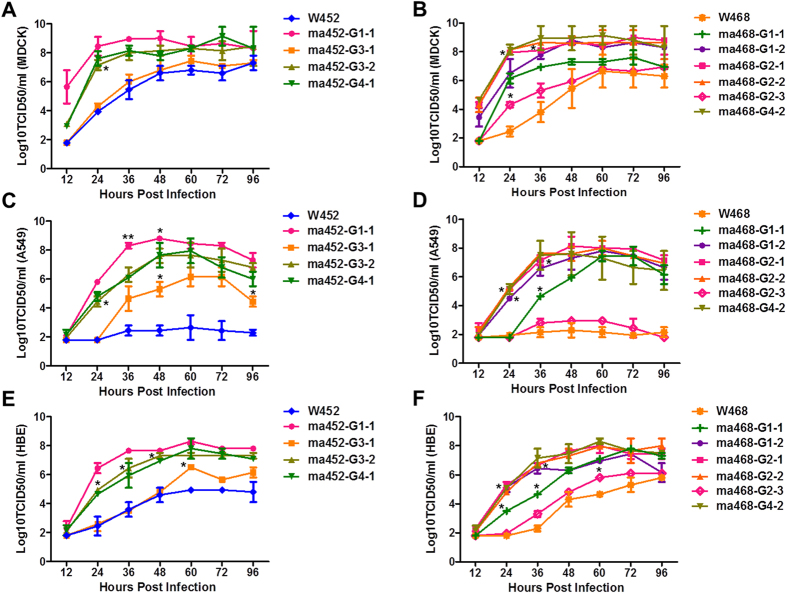
Replication kinetics of the mouse-adapted H5N8 variants *in vitro*. Cells were infected with an MOI of 10^−4^ for MDCK cells (**A** and **B**) or an MOI of 10^−3^ for A549 (**C** and **D**) or HBE (**E** and **F**) cells by using W452 or W468 viruses and their mouse-adapted (ma) strains. Cell culture supernatants were collected at 12, 24, 36, 48, 60, 72, and 96 hpi, titred in MDCK cells, and calculated as the log_10_ TCID_50_/mL. The limit of virus detection was 1.8 log_10_ TCID_50_/ml. Error bars indicate SEMs, which were determined from 3 independent experiments. *P < 0.05, **P < 0.005 compared with the values for wild-type or single mutants (ma452-G3-1, ma468-G1-1, and ma468-G2-3 viruses).

The *in vitro* replicative properties of the maH5N8 viruses were measured in MDCK and human lung/bronchial epithelial cell lines (A549 and HBE) to determine whether they exhibited a growth advantage in mammalian cells. Both wild-type viruses demonstrated minimal growth in all cell lines compared to the ma viruses (Fig. 2). The ma452 G1-1, which carries 9 mutations including the well-known mammalian adaptive markers Q591K and D701N in PB2, exhibited the highest viral growth among the ma452 viruses in all cell lines (Fig. 2A,C and E). Although ma452 G3-1 virus, carrying 1 mutation (D701N in PB2) showed a significantly increased viral titre in A549 cells compared to the wild-type virus, it demonstrated the lowest growth among the ma452 viruses in all cell lines (Fig. 2A,C and E). However, 2 mutations (Q556R in PA and K475N in HA) in combination with PB2 D701N (ma452 G3-2) conferred dramatically improved viral growth to the W452 virus in all cells, including human lung/bronchial epithelial cells (Fig. 2A,C and E). All of the ma468 viruses (G1-2, G2-1, G2-2, and G4-2) carrying multiple mutations, including PB2 E627K, demonstrated higher viral growth than did those carrying a single mutation (G1-1 and G2-3) in all cell lines (Fig. 2B,D and F). Although the single mutant (PB2 E627K) ma468 (G1-1) virus showed significantly higher viral growth than did wild-type viruses in all cell lines, it could not exceed the growth of ma468 viruses carrying multiple mutations (Fig. 2B,D and F). Notably, PB1 P708S or PA T97I in combination with PB2 E627K synergistically increased the viral replicative properties in cell lines, including human lung/bronchial cells (Fig. 2B,D and F). In addition, PB1 P708S alone conferred a significant replicative advantage in MDCK and HBE cells (Fig. 2B and F). Collectively, these results demonstrate that individual mutations and combinations thereof in the maH5N8 viruses significantly and synergistically increased viral replicative properties *in vitro*, including in human lung/bronchial cells.

### Effects of the amino acid substitutions in polymerase genes on viral polymerase activity

**Figure 3:**
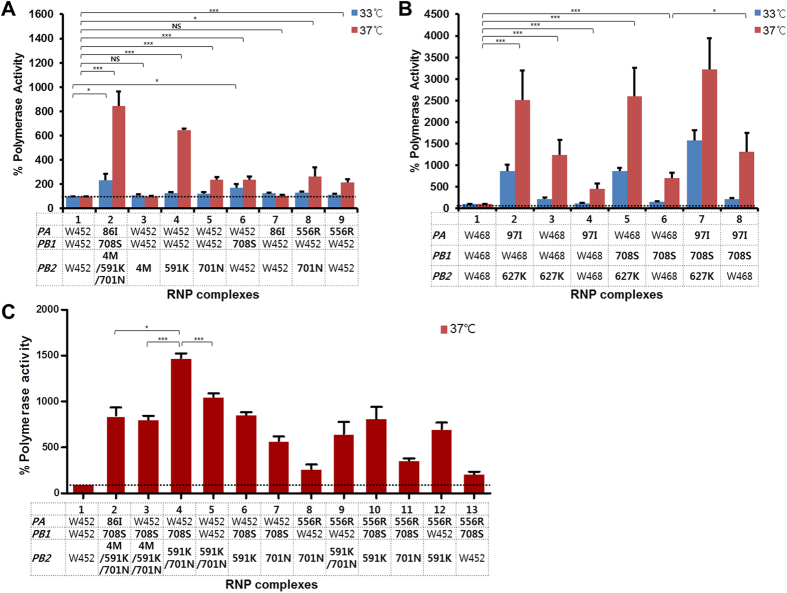
Activity of the polymerase complexes in mouse-adapted H5N8 variants in human cells. The replication and transcription activity levels of reconstituted viral polymerase complexes were measured. The PA, PB1, and PB2 genes of wild-type H5N8 viruses were substituted with polymerase subunits carrying mutations identified during mouse adaptation. The polymerase activity levels of ma452 variants and their single mutants (**A**), ma468 variants and their single mutants (**B**), and recombinant ma452 variants (**C**) were determined using a luciferase-based minigenome reporter assay in 293T cells. Activity values shown are the means of at least 3 assays. The bold formatting of mutations indicates addition of amino acid changes in the RNP complex. Asterisks indicate *p*-values (*P < 0.05; ***P < 0.0001) representing a statistically significant difference. Error bars indicate the SDs of the means.

We found several mutations in the viral polymerase complex of maH5N8 viruses, some of which significantly enhanced viral growth in human lung/bronchial epithelial cells. Thus, to determine whether the mutations in polymerase genes altered H5N8 polymerase activity in human cells, we conducted a minigenome assay using a luciferase-reporter system^24^. Mutations in polymerase genes were introduced, both singly and in combination, into the W452 and W468 viruses. The polymerase complex of the ma452 G1-1 virus carrying I4M, Q591K, and D701N mutations in PB2; P708S in PB1; and M86I in PA (lane 2 in Fig. 3A) showed the highest activity (8.3-fold increase compared to wild type) at both 33 °C and 37 °C among the recombinant ma452 viruses (Fig. 3A). Although 2 single mutations, PB2 I4M and PA M86I, did not affect polymerase activity, the others (PB2 Q591K, D701N, and PA Q556R) increased activity more than 2 fold compared to W452 at 37 °C (Fig. 3A). The ma452 G1-1 virus’ polymerase complex and PB1 P708S single mutant significantly enhanced activity in a human cell line at 33 °C (lanes 2 and 6 in Fig. 3A). We found three mutations in the ma468 viruses (PB2 E627K, PB1 P708S, and PA T97I), and each enhanced polymerase activity more than 4 fold compared to that of W468 virus at 37 °C (Fig. 3B). In addition, double and triple combinations of these mutations synergistically increased polymerase activity compared to that induced by single mutations at 37 °C (Fig. 3B). Viruses carrying the PB2 E627K mutation exhibited dramatically enhanced polymerase activity when combined with the PA T97I and/or PB1 P708S mutation compared to W468 and single mutants at both 33 and 37 °C (lanes 1-6 in Fig. 3B). Different combinations of polymerase mutations derived from ma452 conferred different activities in the ma452 polymerase complex (Fig. 3C). Combinations of multiple mutations generally resulted in synergistic increases in polymerase activity in the ma452 background, though the combination of PB2 I4M with other mutations resulted in significantly decreased activity (lanes 2–4 in Fig. 3C). The mutations arose during mouse adaptation did not enhance polymerase activity in chicken embryonic fibroblasts compared to that of wild-type H5N8 viruses (see Supplementary Fig. S2). These mutations in the polymerase complex of maH5N8 viruses have the potential to synergistically enhance replication in human cells.

### Enhanced virulence, replication, and systemic dissemination of mouse-adapted H5N8 viruses in mice

**Figure 4:**
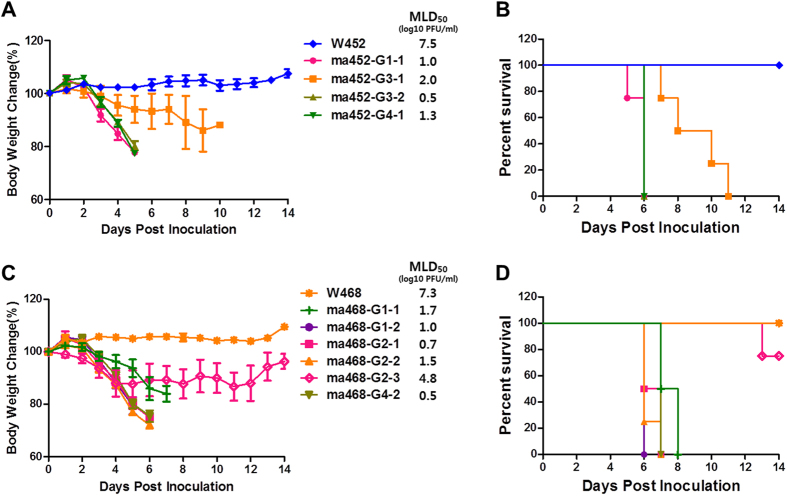
Virulence of the mouse-adapted H5N8 variants in mice. Five-week-old female BALB/c mice were inoculated i.n. with 30 μL of 10^4.0 ^PFU/mL viruses. Weight changes (**A** and **C**) and survival (**B** and **D**) were monitored for as long as 14 dpi. In **A** and **C**, the 50% lethal doses in mice were determined in 10-fold serial-dilution groups (30 μL of 10 to 10^8.0 ^PFU/mL). The MLD_50_ values are shown next to each virus.

To investigate whether the mutations in the maH5N8 viruses alter virulence and replicative properties *in vivo*, we compared the 50% mouse lethal dose (MLD_50_), survival, and viral titres in multiple tissues of mice infected with all viruses, including the wild-type viruses. Groups of mice were i.n. inoculated with 30 μL of 10^4.0 ^PFU/mL, and survival and weight change were monitored for 14 dpi. Infection with any of the ma452 viruses resulted in 100% mortality with robust weight loss, and infection with the W452 virus resulted in 100% survival with no weight loss (Fig. 4A and B). The ma452 viruses carrying mutations in combination with PB2 D701N (i.e., G1–1, G3-2, and G4-1) caused rapid weight loss and mortality in the infected mice within 5 dpi, compared to that of the G3-1 virus carrying the PB2 D701N single mutation, indicating the potential roles of the additional mutations in viral virulence (Fig. 4A and B). Similarly, infections with ma468 viruses, except ma468 G2-3, carrying the PB1 P708S single mutation resulted in 100% mortality with robust weight loss (Fig. 4C and D). However, the ma468 G2-3 virus caused greater weight loss and decreased survival than did W468. To confirm whether the virulence caused by the mutations were mouse age and/or strain-specific, groups of 7-week-old female C57BL/6 mice were infected with equal amounts of viruses as used above. The patterns of survival and weight loss of the infected C57BL/6 mice were similar to that shown in the BALB/c mice, (see Supplementary Fig. S3) suggesting that the mutations can cause high virulence in broad mice strains. The MLD_50_ values of maH5N8 viruses were used as proxies for virulence. Groups of mice were i.n. inoculated with 30 μL of 10-fold serially diluted viruses in a range from 10^1.0^ to 10^8.0 ^PFU/mL (in MDCK cells), and survival and weight loss were monitored for 14 dpi. The maH5N8 viruses showed increased virulence up to the MLD_50_ value of 10^0.5 ^PFU/mL compared to wild-type H5N8 viruses (MLD_50_- 10^7.5^ and 10^7.3 ^PFU/mL of W452 and W468, respectively, *p* < 0.05) (Fig. 4). Combinations of mutations in maH5N8 viruses resulted in lower MLD_50_ values than in the single mutants, indicating that other mutations, including PB1 P708S, PA T97I, and Q556R, contribute to viral virulence, as do PB2 E627K and D701N in mice (Fig. 4). The novel mutation PB1 P708S alone (ma468 G2-3) was associated with increased virulence compared to wild-type W468 (MLD_50_, 10^7.3^ *vs* 10^4.8 ^PFU/mL, respectively, *p* < 0.05) (Fig. 4C), with increased *in vitro* growth and polymerase activity in human cells (Figs 2 and 3).

**Figure 5:**
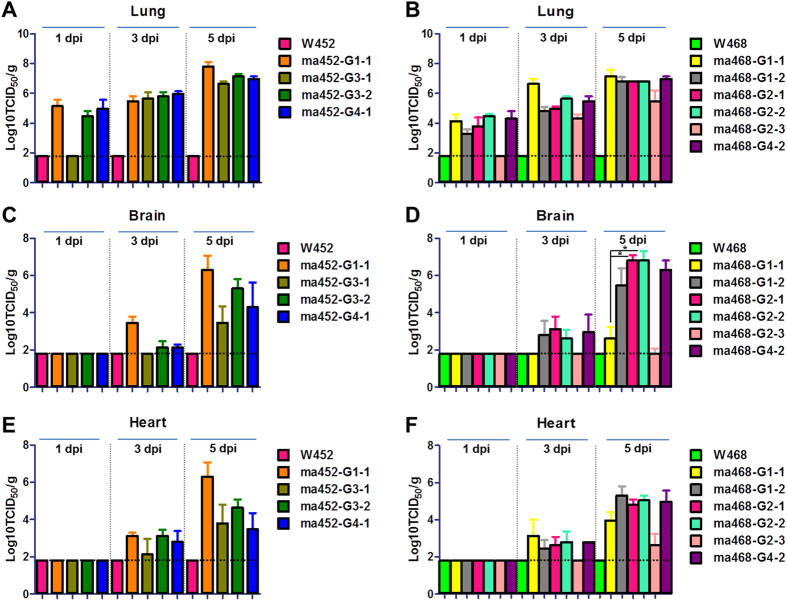
Impact of the adaptive mutations on H5N8 growth in multiple mouse tissues. Five-week-old female BALB/c mice were inoculated i.n. with 30 μL of 10^4.0 ^PFU/mL viruses. Tissue samples of lung (**A** and **B**), brain (**C** and **D**), and heart (**E** and **F**) from 3 ma452- or ma468-infected mice were collected at 1, 3, and 5 dpi. Viral titres were determined in MDCK cells using the Reed-Muench 50% endpoint method and are expressed as the log_10_ TCID_50_/g. *P < 0.05. The limit of virus detection was 1.8 log_10_ TCID_50_/g. Additional data are presented in Supplementary Figure S4.

The common characteristic of HPAI viruses is systemic infection with broad tissue tropism, beyond the primary target organ (i.e., the lung in a mammalian host). Previous studies have demonstrated that H5N8 viruses, including W452, failed to replicate in multiple organs of mammalian hosts, including mice^29^,^30^. Therefore, to investigate whether adaptive mutations confer a replicative advantage in multiple organs (i.e., the lung, brain, heart, kidney, spleen, and colon), we i.n. inoculated groups of mice with 30 μL of 10^4.0 ^PFU/mL. Next, we harvested those tissues at 1, 3, and 5 dpi for viral titration. Among ma452 viruses, ma452 G1-1 exhibited the highest replication in most organs tested (Fig. 5A,C and E, and see Supplementary Fig. S4). Although ma452 G3-1, which carries PB2 D701N, replicated in various organs, it showed delayed growth in all organs, including the lung, compared to other ma452 viruses, and the lowest viral titre in most organs. However, additional mutations, such as PA Q556R and/or K475N (G3-2), enhanced the growth of viruses carrying the PB2 D701N mutation in most organs (Fig. 5A,C and E, and see Supplementary Fig. S4). The single-mutant (PB2 E627K) ma468 G1-1 virus demonstrated similar viral titres in the lung compared with those of other ma468 viruses, but the growth in the brain was substantially impaired (Fig. 5B,D and F, and see Supplementary Fig. S4). Each mutation, PA T97I and PB1 P708S (ma468 G1-2 and G2-1, respectively), in combination with the PB2 E627K mutation, significantly augmented viral titres in the brain (Fig. 5D). Although the single PB1 P708S mutant (ma468 G2-3) showed the lowest viral titres in the lung among ma468 viruses and traces of infectious viruses in the brain and heart (Fig. 5D and F), this newly identified mutation significantly increased viral infectivity compared to that of W468 virus in the mouse lung (5.5 *vs* < 1.7 log_10_ TCID_50_/g) (Fig. 5B,D and F). Because no detectable viral titres of wild-type H5N8 viruses were observed, even in the lungs of mice infected with 30 μL of 10^4.0 ^PFU/mL (Fig. 5 and see Supplementary Fig. S4), we confirmed the systemic dissemination of wild-type H5N8 viruses at a high-inoculation dosage (30 μL of 10^7.0 ^PFU/mL) in mice. Although we detected traces of infectious W452 and W468 viruses in the brain and heart, respectively, most virus infections were limited to the lungs (see Supplementary Fig. S5). To additionally confirm the viral virulence in the host, levels of pro-inflammatory cytokines including *IFN-a, IL-2, IL-6*, and *TNF-a* were measured using whole lungs collected at 1 and 3 dpi. Most of the cytokines tested were more up-regulated in mice lungs infected with high virulent mouse-adapted viruses when compared to those of wild type viruses (see Supplementary Fig. S6). Collectively, the mutations that arose during mouse adaptation of H5N8 virus conferred dramatic enhancement of virulence compared to that of their parental viruses and the capability for systemic dissemination at low-inoculation dosage.

### The PB1 P708S single-mutant virus outgrows wild-type H5N8 in human lung epithelial cells

**Figure 6:**
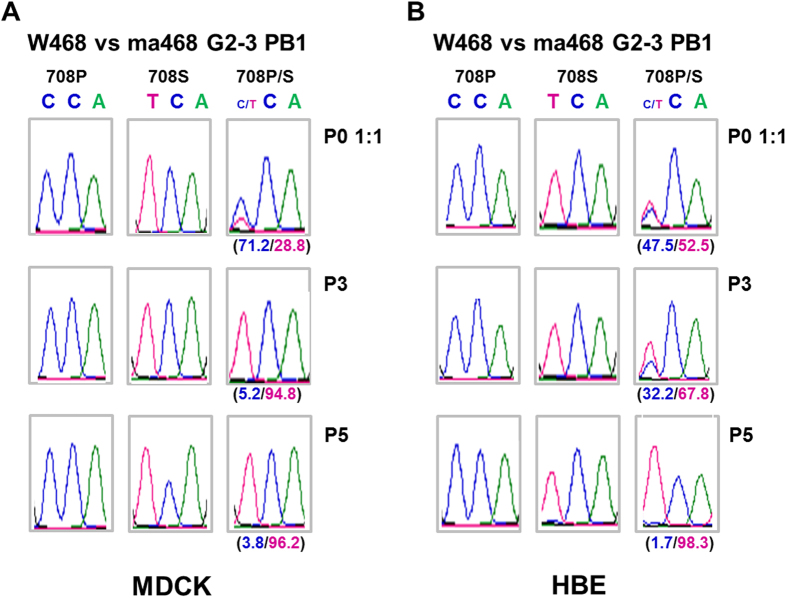
Competitive growth of wild-type H5N8 virus and the PB1 P708S variant virus in mixed cultures. A 1:1 mixture of wild-type H5N8 and the PB1 P708S variant was inoculated into MDCK (**A**) or HBE (**B**) cells at an MOI of 0.01, and equal doses of individual viruses were included as controls. The supernatants at 72 hpi were sequentially passaged 5 times. Viral RNA from P0, P3, and P5 was extracted from supernatants at 72 hpi, and the sequence of the PB1 gene was analysed. The sequence of WT PB1 708P is CCA, and that of the PB1 708 S variant is TCA. The experiments were repeated twice with similar results. One chromatogram is shown. The single nucleotide polymorphism analysis at PB1 708 of the mixed W468 and ma468 G2-3 viruses was depicted in each passage as a percentage ratio. The percentage ratio with blue and red indicate C and T, respectively.

Viral evolution involves genetic selection from the mutation pool in response to host adaptation. Thus, to investigate whether the PB1 P708S single mutant has a selective advantage over the wild-type virus, we inoculated an even mixture of the 2 viruses into MDCK cells and HBE cells and then sequentially passaged those viruses. We used the individual viruses as controls. Sequence analysis of P0, P3, and P5 viruses revealed that the PB1 P708S mutant had outgrown W468 within 5 passages in both cell lines, while the individual viruses retained their intrinsic residues (Fig. 6). This result indicates that the PB1 P708S mutation in the H5N8 virus confers a selective advantage in human lung epithelial cells.

## Discussion

Overcoming the host-restriction barrier of AIVs in a mammalian host requires adaptive markers that facilitate efficient interaction between the virus and host factors and production of viral progenies. Although not all AIVs have become established in humans, H5 and H7 strains have been introduced, have had opportunities to adapt to this new host, and are now common. Many studies have demonstrated how AIVs overcome host-restriction barriers on the molecular level. Adaptive markers are found predominantly in the viral polymerase complex, including E627K, D701N, K526R, T271A, Q591K, and G590S/Q591R motifs in PB2 and T97I in PA^17^,^18^,^19^,^20^,^22^,^24^,^25^,^32^. In general, those adaptive mutations confer an advantage to viral replication; therefore, they are often associated with severe clinical outcomes. However, not all mutations improve AIV adaptation in mammalian hosts, and some are strain and/or host specific. Therefore, it is essential to investigate mammalian-adaptive markers in diverse virus strains, particularly strains that have been introduced into humans and have pandemic potential. We found that the newly emerged HPAI H5N8, the only strain to cross from Eurasia to America^5^,^33^, can rapidly attain established and novel virulence markers during adaptation to the mammalian host, underscoring the role of genetic plasticity in this process. Furthermore, combinations of the mutations synergistically enhanced replication, virulence, and systemic dissemination *in vitro* and/or *in vivo*.

Many mutations have been reported in the viral polymerase complex, which most likely plays a crucial role in host adaptation^32^. We observed a variety of mutations in the polymerase subunits of H5N8 viruses, including PB2 I4M, Q591K, E627K, and D701N; PB1 P708S; and PA M86I, T97I, and Q556R. Strains W452 and W468 independently developed adaptive mutations, though they share 98.9% to 99.7% genetic homology among 8 gene segments (see Supplementary Table S1). W452, which was isolated during the first outbreak in 2014 in South Korea, consistently acquired PB2 D701N, and W468, which was isolated during the second wave in 2015, showed the conserved PB2 E627K substitution. Similarly, two HPAI H5N2 viruses were noted to acquire distinct mammalian adaptive markers, either PB2 E627K^34^ or D701N^35^ in mice. A recent surveillance studies suggested that H5N8 viruses isolated during each outbreak were phylogenetically distinct^31^,^36^, suggesting that minor genetic differences between the 2 viruses potentially resulted in distinctive selection of adaptive markers; however, further study will be needed to test these hypotheses.

The well-characterized mammalian adaptive markers PB2 E627K and D701N substantially enhanced viral growth and polymerase activity *in vitro* and virulence *in vivo* in an H5N8 background, which was in agreement with an earlier study^32^. Moreover, we observed that combinations of mutations, including PB1 P708S, PA T97I, and/or Q556R conferred synergistic effects on H5N8, suggesting that H5N8 exploits genetic diversity to adapt to mammalian hosts. Whether the PB2 Q591K mutation increases viral growth and/or virulence was not confirmed because the single mutant was not selected by plaque purification. Therefore, it must be further evaluated. Given the reported adaptive effects of this substitution in mammalian hosts^37^,^38^ and the fact that the variant carrying this mutation showed the highest relative replication rate and dramatically increased polymerase activity, we assume that PB2 Q591K probably contributes to viral growth and/or virulence in a mammalian host. Substitution of PB2 Q591 into basic residues (R/K) have been suggested as an alternative substitution for PB2 E627K^38^,^39^, much like PB2 D701N/E627K. However, natural selection of a PB2 Q591K/D701N double mutant during mammalian adaptation in this and another study^35^ suggests that 2 virulence markers, PB2 K591/N701, could coexist in HPAI H5. Amino acid position I4 in PB2 is highly conserved among influenza viruses and is involved in direct interaction with the C terminal region of PB1^40^. The rates of I4D and I4M mutations defined in this study were associated with significantly reduced polymerase activities. Although PB2 I4M was selected for by the ma452 G1-1 and inhibited viral polymerase activity, the virus showed the highest replication rate and virulence.

The novel adaptive marker PB1 P708S confers a biological advantage to the H5N8 virus in mammalian hosts and may be strain specific due to the low percentage of S708 in field isolates (see Supplementary Table S3). Most (>99.9%) human influenza viruses and AIVs carry PB1 P708 (7 viruses carry S708), suggesting that the substitution is specifically acquired in a gene constellation similar to that seen in H5N8 during mammalian adaptation. The residue P708 in the C-terminal region of PB1 is involved in the interaction with the hydrophobic residues in the N-terminal region of PB2^40^. The P708S substitution may disturb hydrophobic interactions between the residues PB1 F696 and F700 and P708, and PB2 V25 (see Supplementary Fig. S7). In addition, the substitution may affect the endonuclease activity of PA by altering the PB1 loop structure that has been reported previously as different conformations^40^,^41^ (see Supplementary Fig. S7). Future structural biological studies should clarify the role of the PB1 P708S substitution in mammalian adaptation.

PA carries a viral endonuclease that is important to host cap snatching for viral transcription^42^ and is further involved in mammalian adaptation. The 3 mutations identified in the PA gene during mouse adaptation in this study were M86I, T97I, and Q556R. In our previous study, the PA T97I mutation arose during low-pathogenic avian influenza (LPAI) H5N2 adaptation to mice^24^. Other studies^25^,^43^,^44^,^45^ have also observed this mutation in correlation with viral replication and virulence in mammalian hosts. This mutation was associated with significantly and synergistically increased viral growth and polymerase activity in human cells and contributed to increased virulence *in vivo*. Although PA Q556R has been suggested as a mouse-specific substitution^46^, the mutation in the backbone of H5N8 conferred enhanced polymerase activity and potential synergistic effects on viral growth in human cells. Further study will be required to verify the function of PA M86I, but the mutation showed no significant effect on polymerase activity, and I86 is not common in the field strains, suggesting that it was a random mutation.

We also observed several changes in HA, a protein essential to viral attachment and fusion and a determinant of host-receptor specificity^47^. The HA A156T, which was found in ma452 and ma468 viruses, potentially adds an N-glycosylation to the globular head domain of the HA protein. Loss of glycosylation at this site enhances binding to Siaα2, 6Gal, a preferential human receptor, and is relevant to H5N1 virus transmissibility in mammalian hosts^21^,^48^,^49^. Neumann and colleagues further suggested that HPAI H5 viruses, such as Egyptian H5N1, that lack glycosylation at residues 154 to 156 present a far greater pandemic risk. Although an maH5N8 study partly resulted in acquisition of glycosylation at those positions, wild-type H5N8 virus originally lacking the glycosylation could have higher risk potential. However, the A156T change does not alter the avian receptor binding preference of H5N8 virus (see Supplementary Fig. S8), implying the importance of genetic background in determining the role of glycosylation in host-receptor specificity. The HA2 domain is mainly involved in HA fusion, which is caused by acidification in the endosome and facilitates genome delivery into the cytosol^47^. However, mutations in HA of maH5N8 viruses do not alter the viral fusion pH, as measured in a pH-dependent haemolysis assay (see Supplementary Fig. S8). We found a conserved K-to-N substitution at position 475 in the HA gene of ma452 viruses, and HA N475 is predominant in H5N8 viruses (>99%), as revealed by single amino acid polymorphism analysis (see Supplementary Table S3). This substitution might restore the genetic constellation.

An additional advantage of using the mammalian host–adaptation method to identify virulence markers is the capacity to estimate how rapidly the emerging virus acquires a virulence phenotype in the host. The HPAI H5N8 virus caused severe clinical disease in mice but only at high-inoculation dosages^29^,^30^. However, the H5N8 viruses used here demonstrated lethal pathogenicity within 5 dpi in 2 sequential passages at low-inoculation dosage, while generally, HPAI and LPAI viruses demonstrated detectably increased virulence after 5 or more passages in mice^24^,^50^,^51^,^52^,^53^. The rapid acquisition of virulence by the H5N8 viruses implies increased pandemic potential, especially with worldwide spread and rapid evolution^4^,^54^.

In this study, we used plaque-purified viruses; thus, determining the precise roles of all individual changes was not possible. Therefore, the roles of single mutations or multiple combinations of the markers need to be determined using reverse genetics to minimize potential confounders in future studies. However, adequate and sequential distribution of the individual mutations in the plaque-purified viruses can facilitate elucidation of their effects. We observed dramatically enhanced virulence of H5N8 in a mammalian host within a short period of time, and its genetic plasticity made it possible to obtain polymorphic virulence markers. However, it is not guaranteed that a virus that has spread globally will stably maintain the genetic background that favours the avian host. Therefore, intensive monitoring of the genetic evolution of H5N8 viruses is essential, and minimizing human contact with avian species infected with H5N8 influenza will help control viral evolution at the frontlines of zoonotic transmission.

## Methods

### Cells and viruses

Human lung adenocarcinoma epithelial (A549) cells and human embryonic kidney (293T) cells were propagated and maintained in Dulbecco’s modified Eagle’s medium (DMEM; Gibco-Invitrogen, Carlsbad, CA) containing 10% foetal calf serum (FCS; Omega Scientific, Tarzana, CA) and 1% antibiotics (penicillin/streptomycin, Gibco-Invitrogen). Madin-Darby canine kidney (MDCK) cells were propagated and maintained in Eagle’s minimal essential medium (EMEM; Lonza, Allendale, NJ) supplemented with 5% FCS and 1% antibiotics. Primary human bronchial epithelial (HBE) cells were purchased from ScienCell Research Laboratories (Carlsbad, CA) and were differentiated as previously described^55^. Cells were incubated at 37 °C in 5% CO_2_ until use.

A/Mallard duck/Korea/W452/2014 (W452, H5N8) and A/Environment/Korea/W468/2015 (W468, H5N8), both HPAI viruses, were isolated from wild bird faecal samples during the first and second waves of an H5N8 outbreak in South Korea, respectively^31^. The viruses were propagated in specific pathogen–free (SPF) 10-day-old embryonated chicken eggs and stored at –80 °C until use. Stock viral titres were determined by plaque assay in MDCK cells and are expressed as PFU/mL. All experiments with HPAI H5N8 viruses were conducted in an enhanced biosafety level three (BSL3+) laboratory approved by the Korea Center for Diseases Control.

### Adaptation of wild-type H5N8 viruses in mice and identification of substitutions in the viral genome

Groups of four 5-week-old female BALB/c mice were anesthetized with an intraperitoneal injection of Zoletil/Xylazine mixture (20 mg/kg) and inoculated i.n. with 30 μL of 10^7.0 ^PFU/mL of wild-type W468 or W452 viruses. The mice were monitored for weight loss and mortality for 5 dpi. At 5 dpi, 4 inoculated mice were euthanized, and their lungs were collected and homogenized (1 g/mL) in cold phosphate-buffered saline (PBS) containing antibiotics (0.1% penicillin/streptomycin). The homogenate was centrifuged at 13000 × *g* for 5 min at 4 °C, and 30 μL supernatant or 1:100 to 10,000 dilutions of supernatant were independently inoculated into 4 naïve mice. After 5 passages, the viruses in the lung homogenates were prepared for plaque purification in MDCK cells. The viruses from lung homogenates were sequenced using the conventional Sanger-sequencing method. Briefly, RNA was extracted from the lung samples using an RNeasy Mini kit (QIAGEN, Valencia, CA). RT-PCR was carried out under standard conditions using influenza-specific primers^56^. Nucleotide sequencing of the amplified products was carried out by a specialized sequencing company (Cosmogenetech co., Korea). The DNA sequences were compiled and analysed using the Lasergene sequence analysis software package (DNASTAR, Madison, WI). All animal experiments were conducted in accordance and adherence to relevant policies on animal handling as mandated in the Guidelines for Animal Use and Care of the Chungbuk National University and the experimental protocol was approved by Institutional Biosafety Committee in Chungbuk National University. The substituted amino acids were identified by comparison to the wild-type H5N8 sequence, and single-nucleotide polymorphisms (SNPs) in the mutants were further analysed by comparison with as many as 27,000 sequences from avian and human isolates using the SNP-analysis function in the Influenza Research Database (http://www.fludb.org/brc/home.spg?decorator=influenza).

### Plaque purification, morphology, and selection

To segregate clones of maAIVs, a standard plaque purification assay was performed. The viruses adapted to the mouse lung were individually prepared in serial 10-fold dilutions of each sample with infection medium. After monolayers of MDCK cells (1 × 10^6^ cells) were maintained in culture in 6-well plates and washed twice with PBS, the cells were infected with 1 mL of serial diluents and incubated at 37 °C in a CO_2_ incubator 1 h for virus absorption. Next, the supernatants were transferred to 0.7% agar containing infection medium, penicillin (1000 units/mL), streptomycin (1000 μg/mL), vitamin solution, 0.5% bovine serum albumin, and 0.1% TPCK-trypsin. After 48 h of incubation at 37 °C in a CO_2_ incubator, 2 to 3 plaques from each mouse-adapted sample were initially picked for purification. Picked plaques were resuspended in 500 μL PBS and propagated in SPF 10-day-old embryonated chicken eggs for 36 h. The cloned viruses were named ma452 G1through G4 and ma468 G1 through G4. The full sequences of cloned viruses were confirmed using conventional Sanger sequencing after RT-PCR, and the viruses associated with high mortality in mice and/or those needed for further study were selected. Viral titres and the morphologies of the selected clones were determined in MDCK cells by fixation with 4% ice-cold formalin and staining with 1% crystal violet for visualization. The average plaque size was calculated as the mean size of 30 or more individual plaques by using ImageJ software.

### *In vitro* viral growth kinetics

Wild-type and mouse-adapted/plaque-purified viruses were inoculated into monolayers of MDCK cells [multiplicity of infection (MOI) of 0.0001] and monolayers of A549 and HBE cells (MOI of 0.01). After incubation at 37 °C for 1 h, the supernatants were replaced with infection medium without TPCK-trypsin. Cell culture supernatants were harvested at 12, 24, 36, 48, 60, 72, and 96 hpi for virus titration. The viral titres of supernatants at each time point were measured in MDCK cells via the TCID_50_/mL method.

### Minigenome assay for polymerase activity

A luciferase activity–based minigenome reporter assay was conducted as described by Song *et al*.^24^. A luciferase-reporter plasmid was constructed by replacing the open–reading frame of enhanced green fluorescent protein (EGFP) in the pHW72-EGFP plasmid with the luciferase gene. Ribonucleoprotein complexes composed of pHW72-Luc, pHW2000-PB2, pHW2000-PB1, pHW2000-PA, and pHW2000-NP (0.1 μg each) were mixed with pCMV–β-galactosidase plasmids, then cotransfected into 293T cells that had been prepared in 24-well plates 24 h previously by using TransIT-LT1 transfection reagent, as directed. After 6 h, the transfection medium was replaced with fresh, complete DMEM. The luciferase activity was measured at 36 h posttransfection. Next, the cells were washed with PBS and lysed for 30 min with 100 μL lysis buffer (Promega, Madison, WI). Cell lysates then were harvested, and luciferase activity was assayed in triplicate using the luciferase assay system. Beta-galactosidase activity was detected for each sample and used for normalization of minigenome expression across samples.

### Viral replication, dissemination, and virulence in mice

Groups of thirteen 5-week-old female BALB/c mice were anesthetized with an intraperitoneal injection of Zoletil/Xylazine mixture (20 mg/kg), inoculated i.n. with 30 μL of 1 × 10^4.0 ^PFU/mL of infectious virus, and monitored daily for weight loss and mortality for 14 days. To determine virus titres in various tissues, 3 mice from each group were euthanized on 1, 3, and 5dpi, and organs (i.e., lungs, spleens, colon, liver, heart, and brains) were harvested. The collected organs were homogenized (1 μg/mL) in cold PBS containing antibiotics (0.1% penicillin/streptomycin). The supernatant was used to measure virus titre by TCID_50_. To determine the MLD_50_, based on PFU/mL of viruses, groups of 4 mice were i.n. inoculated with 10-fold serial dilutions from 10 to 10^7.0 ^PFU/mL. The MLD_50_ was expressed as log_10 _PFU/mL. All 50% tissue culture infectious dose (TCID_50_) and MLD_50_ calculations were performed using the method of Reed and Muench^57^.

### Sequential passage of mixtures of H5N8 (PB1-708P) and (PB1-708S)

W468 (PB1-708P) and ma468 G2-3 (PB1-708S) viruses were evenly mixed and inoculated into MDCK cells (MOI of 0.001) and HBE cells (MOI of 0.01). After incubation at 37 °C for 1 h, the supernatants were replaced with serum-free medium without trypsin, as appropriate for each cell line. The supernatant was harvested after 72 h and sequentially and independently passaged in each cell line 4 more times. Sequential passages of individual viruses were also conducted for comparison. The PB1 genes of viruses from P0, P3, and P5 were sequenced using RT-PCR with a customized primer set (forward primer_PB1-1901F, 5′-ATAAGGAAATTGAGTCCGTAA-3′; reverse primer_PB1-2166R, 5′-GAGGCCATGGTGTCTAGGGCC-3′) containing the amino acid sequence at position PB1 708. Changes in nucleotide peaks were analysed using the Lasergene sequence analysis software package (DNASTAR, Madison, WI). For single nucleotide polymorphism (SNP) analysis, the PCR products of the PB1 fragments from mixed viruses (W468, ma468 G2-3) were analysed by a company specializing in NGS sequencing (GNCBIO Inc., Korea). As briefly described in the methods section, PCR fragments were ligated with Ion P1 adaptor and Ion Xpress ™ Barcode X at both 3′ and 5′ end. The Library preparation was performed according to the manufacturer’s protocol using the Ion 540 kit for S5 sequencing. The sequencing reads were then demultiplexed, quality-trimmed, and filtered using the PRINSEQ. Reads were aligned to the wild-type virus sequence, and the mapped reads were put through the Samtools and Bcftools software programs.

### Statistical analysis

The differences of plaque size, viral titer, survival, and polymerase activity between wild type and mouse-adapted/plaque-purified H5N8 viruses were compared using a t-test or one-way ANOVA. Differences were considered as significant at p < 0.05. The statistical analysis was carried out using GraphPad Prism software (Ver. 5.02).

## Additional Information

**How to cite this article**: Choi, W.-S. *et al*. Rapid acquisition of polymorphic virulence markers during adaptation of highly pathogenic avian influenza H5N8 virus in the mouse. *Sci. Rep.* **7**, 40667; doi: 10.1038/srep40667 (2017).

**Publisher's note:** Springer Nature remains neutral with regard to jurisdictional claims in published maps and institutional affiliations.

## Supplementary Material

Supplementary Information

## Figures and Tables

**Table 1:** Amino acid sequence comparison across wild-type and mouse-adapted/plaque-purified H5N8 viruses.

Virus	PB2				PB1	PA			HA^a^						Mean plaque size^b^ (mm ± SD)	Stock viral titre (PFU/mL)
	4	591	627	701	708	86	97	556	40	156	181	322	395	475		
W452	I	Q	E	D	P	M	T	Q	K	A	S	L	V	K	0.43 ± 0.13	1.1 × 10^8^
ma452 G1-1	M	K	—	N	S	I	—	—	E	T	—	—	I	N	2.15 ± 0.41	3.0 × 10^8^
ma452 G3—1	—	—	—	N	—	—	—	—	—	—	—	—	—	—	0.45 ± 0.15	5.3 × 10^6^
ma452 G3—2	—	—	—	N	—	—	—	R	—	—	—	—	—	N	1.07 ± 0.21	9.7 × 10^7^
ma452 G4—1	—	—	—	N	—	—	—	R	—	—	T	—	—	N	1.03 ± 0.29	3.9 × 10^8^
W468	I	Q	E	D	P	M	T	Q	K	A	S	L	V	N	0.34 ± 0.10	4.8 × 10^8^
ma468 G1—1	—	—	K	—	—	—	—	—	—	—	—	—	—	—	0.33 ± 0.19	2.4 × 10^8^
ma468 G1—2	—	—	K	—	—	—	I	—	—	—	—	—	—	—	0.93 ± 0.24	6.0 × 10^7^
ma468 G2—1	—	—	K	—	S	—	—	—	—	—	—	—	—	—	1.45 ± 0.46	1.9 × 10^8^
ma468 G2—2	—	—	K	—	S	—	—	—	—	T	—	—	—	—	1.62 ± 0.29	2.2 × 10^8^
ma468 G2—3	—	—	—	—	S	—	—	—	—	—	—	—	—	—	0.35 ± 0.12	2.2 × 10^8^
ma468 G4—2	—	—	K	—	S	—	—	—	—	T	—	P	—	—	1.60 ± 0.27	2.8 × 10^8^
Gyr/WA/41088—6^c^	—	—	—	—	—	—	—	—	—	—	—	—	—	—	NA	NA

^a^N3 numbering.

^b^Additional data on the plaque morphology of H5N8 viruses are shown in Supplementary Figure S1.

^c^Reference sequence, A/Gyrfalcon/Washington/41088-6/2014 (H5N8).

**Abbreviations:** A, alanine; D, aspartic acid; E, glutamic acid; HA, haemagglutinin gene; I, isoleucine; K, lysine; L, leucine; M, methionine; ma, mouse-adapted; N, asparagine; P, proline; PA, polymerase acidic; PB1, polymerase basic 1; PB2, polymerase basic 2; PFU, plaque-forming unit; Q, glutamine; R, arginine; S, serine; SD, standard deviation; T, threonine; V, valine.
