# Supplementary material for: Rapid acquisition of polymorphic virulence markers during adaptation of highly pathogenic avian influenza H5N8 virus in the mouse
Source: Sci Rep. 2017 Jan 17;7:40667. doi: 10.1038/srep40667 (PMC5240553; doi:10.1038/srep40667)
Supplement: Supplementary Information [file srep40667-s1.pdf]

Supplementary Materials for:

Rapid acquisition of polymorphic virulence markers during adaptation of highly pathogenic avian influenza H5N8 virus in the mouse

Won-Suk Choi, Yun Hee Baek, Jin Jung Kwon, Ju Hwan Jeong, Su-Jin Park, Young-il Kim, Sun-Woo Yoon, Jungwon Hwang, Myung Hee Kim, Chul-Joong Kim, Richard J. Webby, Young Ki Choi, Min-Suk Song

Correspondence to: **songminsuk@chungbuk.ac.kr**

choiki55@chungbuk.ac.kr

richard.webby@stjude.org

Supplementary Figures

Supplementary Figure S1

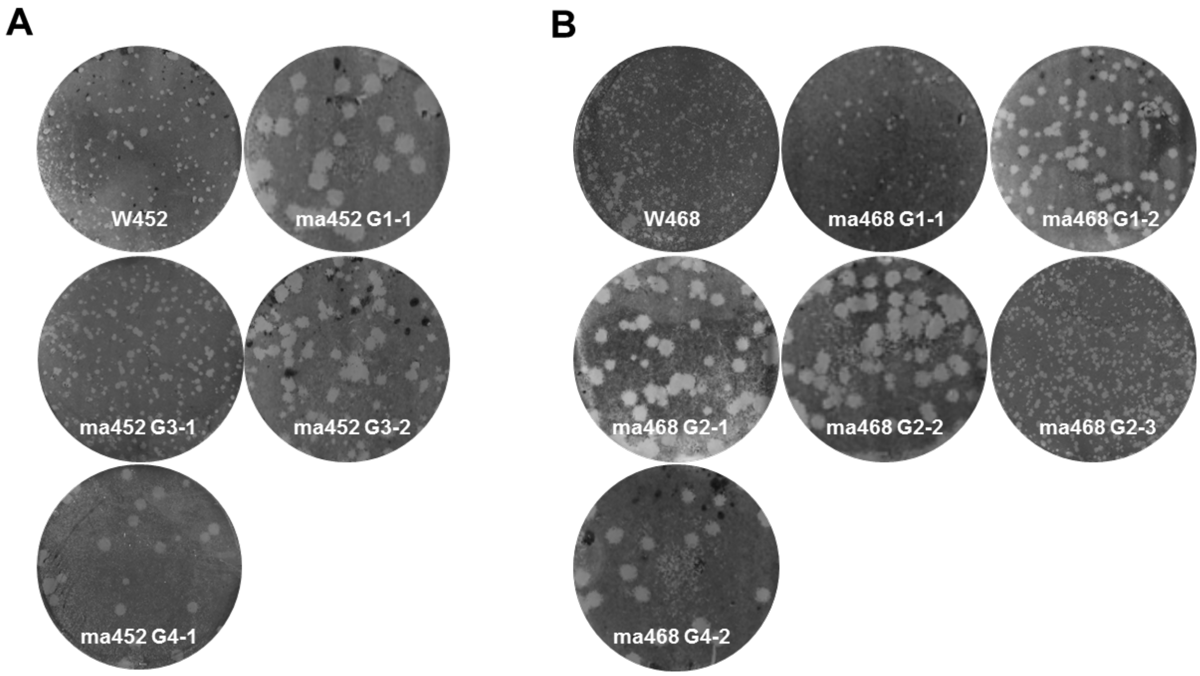

Supplementary Figure S1. Plaque morphologies of wild-type and mouse-adapted, plaque-purified H5N8 viruses in MDCK cells. Plaque morphologies of W452 (**A**) and W468 (**B**) and their mouse-adapted colonies were observed in MDCK cells. Group numbers indicate purified plaques (clones) from the independently passaged groups (G1-G4-1 through 3).

Supplementary Figure S2

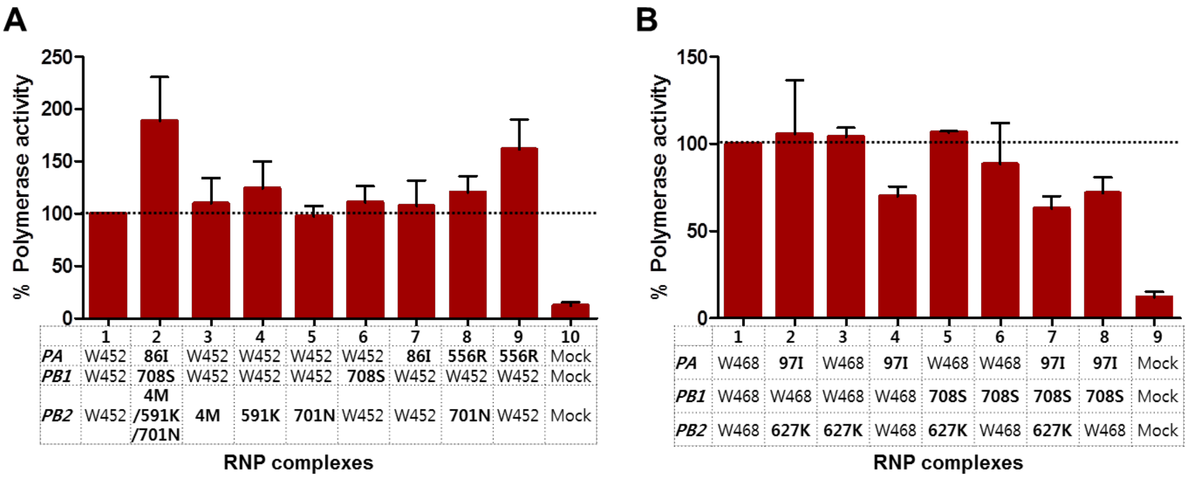

Supplementary Figure S2. Activity of the polymerase complexes of mouse-adapted H5N8 variants in chicken embryonic fibroblasts. The replication and transcription activity levels of reconstituted viral polymerase complexes in which the PA, PB1, and PB2 genes of wild-type H5N8 viruses were substituted with polymerase subunits carrying mutations that arose during mouse adaptation. The polymerase activities of ma452 variants and their single mutants (**A**) and ma468 variants and their single mutants (**B**) were determined in a luciferase-based minigenome reporter assay in chicken embryonic fibroblasts. Activity values shown are the means of values from at least 3 assays. Error bars indicate standard deviations of the mean.

Supplementary Figure S3

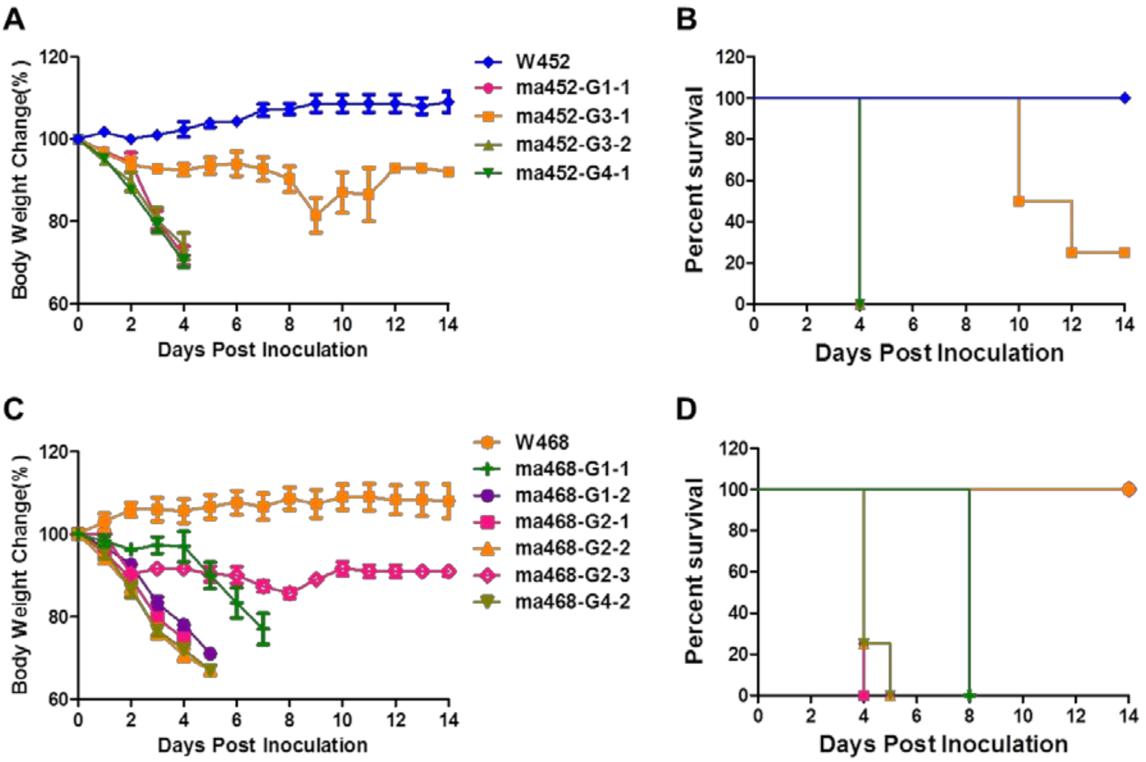

Supplementary Figure S3. Virulence of the mouse-adapted H5N8 variants in 7-week-old C57BL/6 mice. Seven-week-old female C57BL/6 mice were inoculated i.n. with 30 μL of $10^{4.0}$ PFU/mL viruses. Weight changes (**A** and **C**) and survival (**B** and **D**) were monitored for as long as 14 dpi.

Supplementary Figure S4

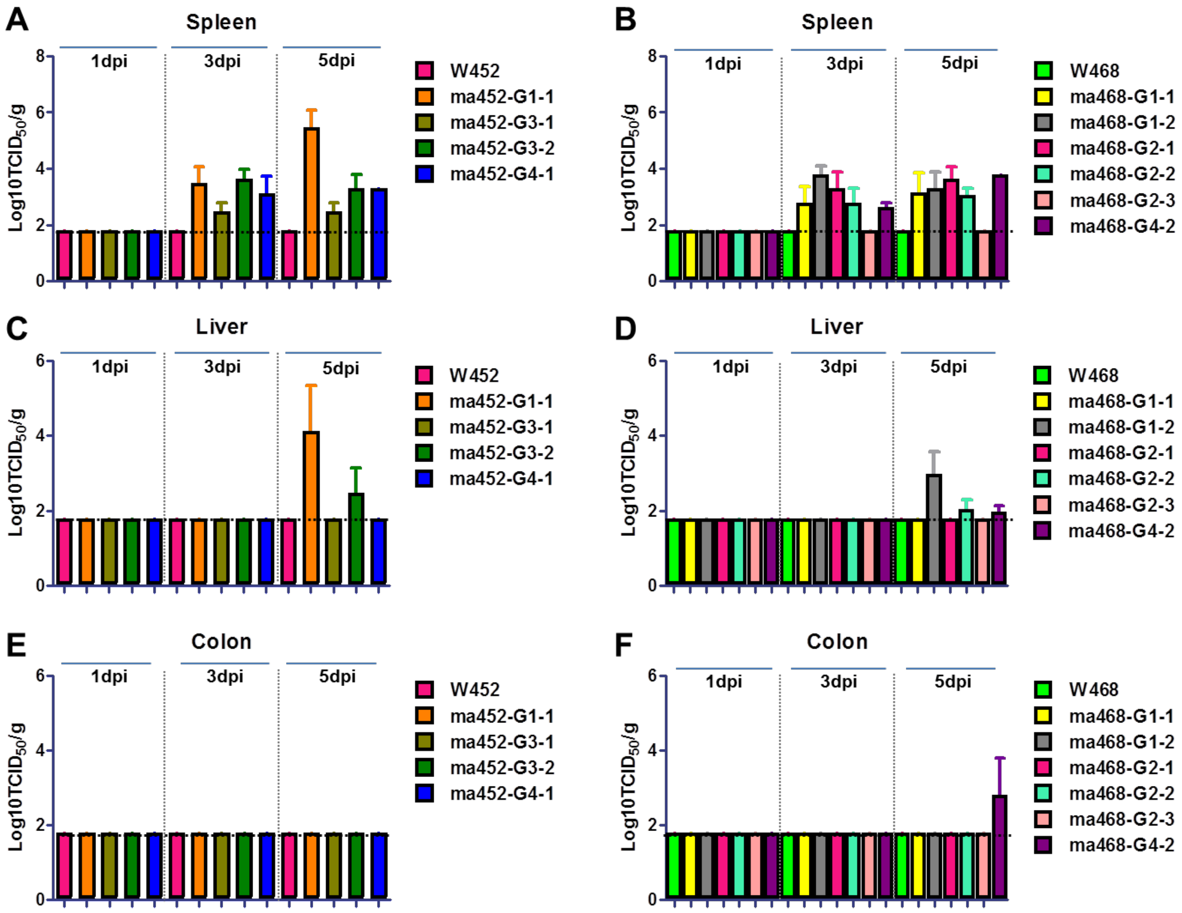

Supplementary Figure S4. Impact of the adaptive mutations on H5N8 growth in multiple mouse tissues. Five-week-old female BALB/c mice were inoculated i.n. with 30 μL of 10^4 PFU/mL of viruses. Tissue samples, including spleen (**A** and **B**), liver (**C** and **D**), and colon (**E** and **F**), were collected from 3 mice at 1, 3, and 5 dpi. Viral titres were determined in MDCK cells by using the Reed-Muench 50% endpoint method[1] and are expressed as log10 $TCID_{50}$/g. The limit of virus detection was 1.8 log_{10} $TCID_{50}$/g. Additional data are presented in Figure 5.

Supplementary Figure S5

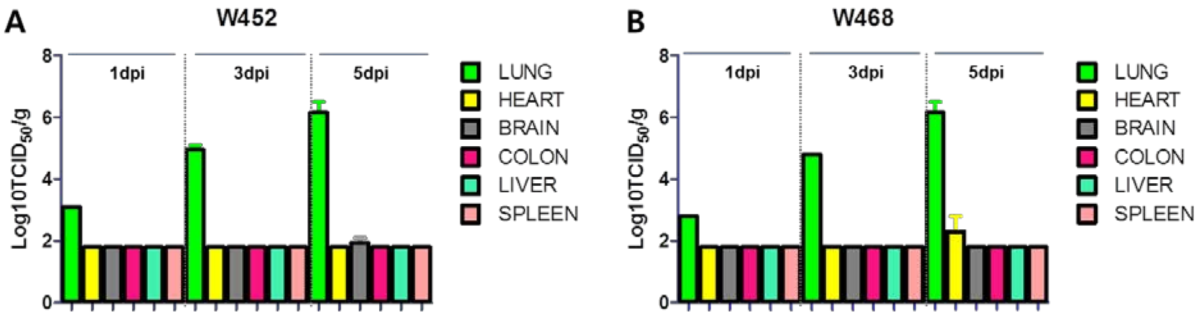

Supplementary Figure S5. Replicative properties of wild-type H5N8 viruses in multiple tissues of mice at a high-inoculation dose. Five-week-old female BALB/c mice were inoculated i.n. with 30 μL of 10^7 PFU/mL of wild-type H5N8 viruses W452 (**A**) and W468 (**B**). Tissue samples, including spleen, liver, and colon, were collected from 3 mice at 1, 3, and 5 dpi. Viral titres were determined in MDCK cells by using the Reed-Muench 50% endpoint method[1] and are expressed as log10 TCID$_{50}$/g.

Supplementary Figure S6

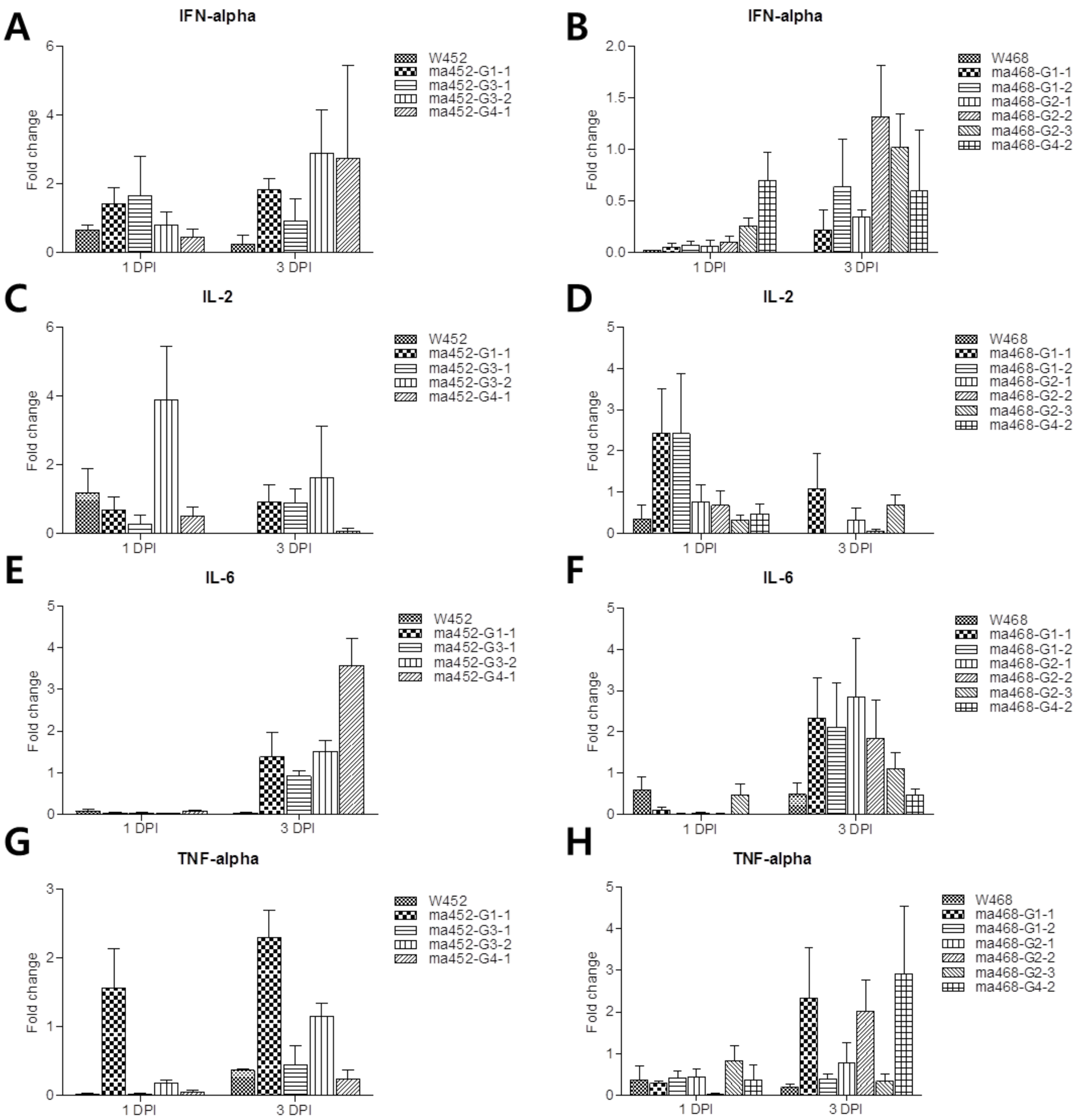

Supplementary Figure S6. The changes of pro-inflammatory cytokines in virus-infected mice lungs. Mice lungs infected with wild type and mouse-adapted viruses were harvested at 1 and 3 dpi; RNA levels of pro-inflammatory cytokines including *INF-a* (A and B), *IL-2* (C and D), *IL-6* (E and F), and *TNF-a* (G and H) were measured by a relative quantitative real-time PCR method. Error bar indicates a standard error mean of three lung samples

Supplementary Figure S7

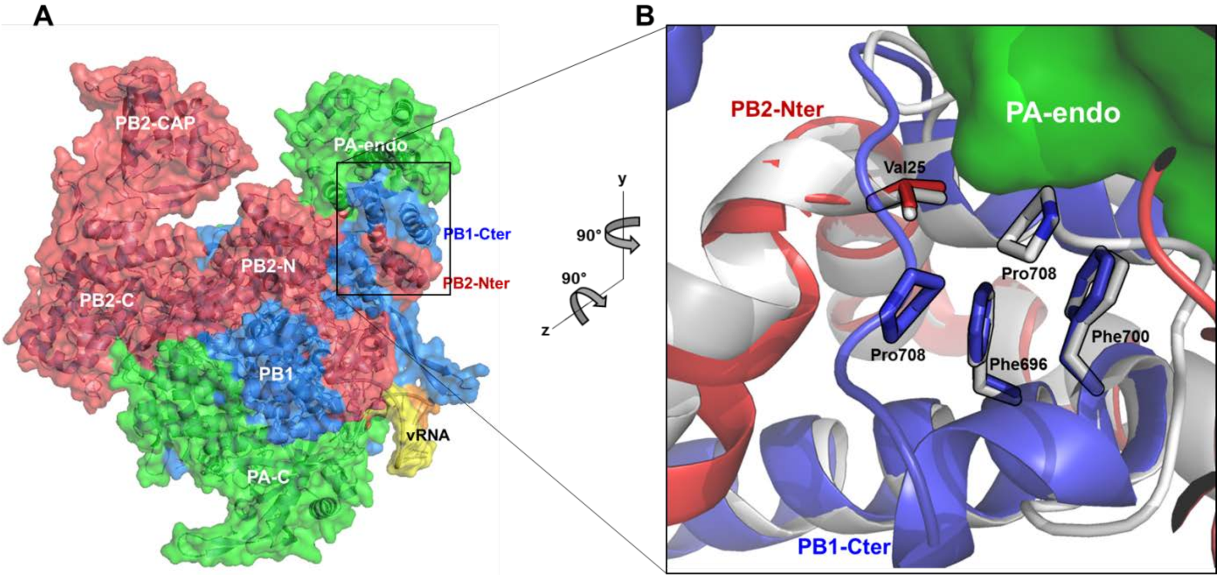

Supplementary Figure S7. The location of the PB1 P708 in the structure of polymerase complex. (A) Surface view of the structure of bat influenza A polymerase complex (Protein Data Bank accession code 4WSB). PA, PB1 and PB2 are shown in green, blue and red, respectively. The flexible region that may undergo local conformational change by PB1 P708S substitution is indicated by the outlined box. (B) Close-up view of the potential movable loop. The structure of the PB1-PB2 interaction domain (Protein Data Bank accession code 2ZTT), which is shown in gray, is superimposed onto the structure of the bat influenza A polymerase complex. The residues involved in the local hydrophobic interaction between PB1-Cter and PB2-Nter are indicated. PB1 P708 of bat influenza A polymerase complex contributes to the formation of the loop structure distinctively different from that of PB1-PB2 interaction domain structure (Protein Data Bank accession code 2ZTT). PB2-CAP, PB2 cap-binding domain; PB2-C, C-terminal domain of PB2; PB2-N, N-terminal domain of PB2; PB2-Nter, N-terminal region of PB2; PA-endo, N-terminal endonuclease domain of PA; PA-C, C-terminal domain of PA; PB1-Cter, C-terminal region of PB1; vRNA, viral RNA.

Supplementary Figure S8

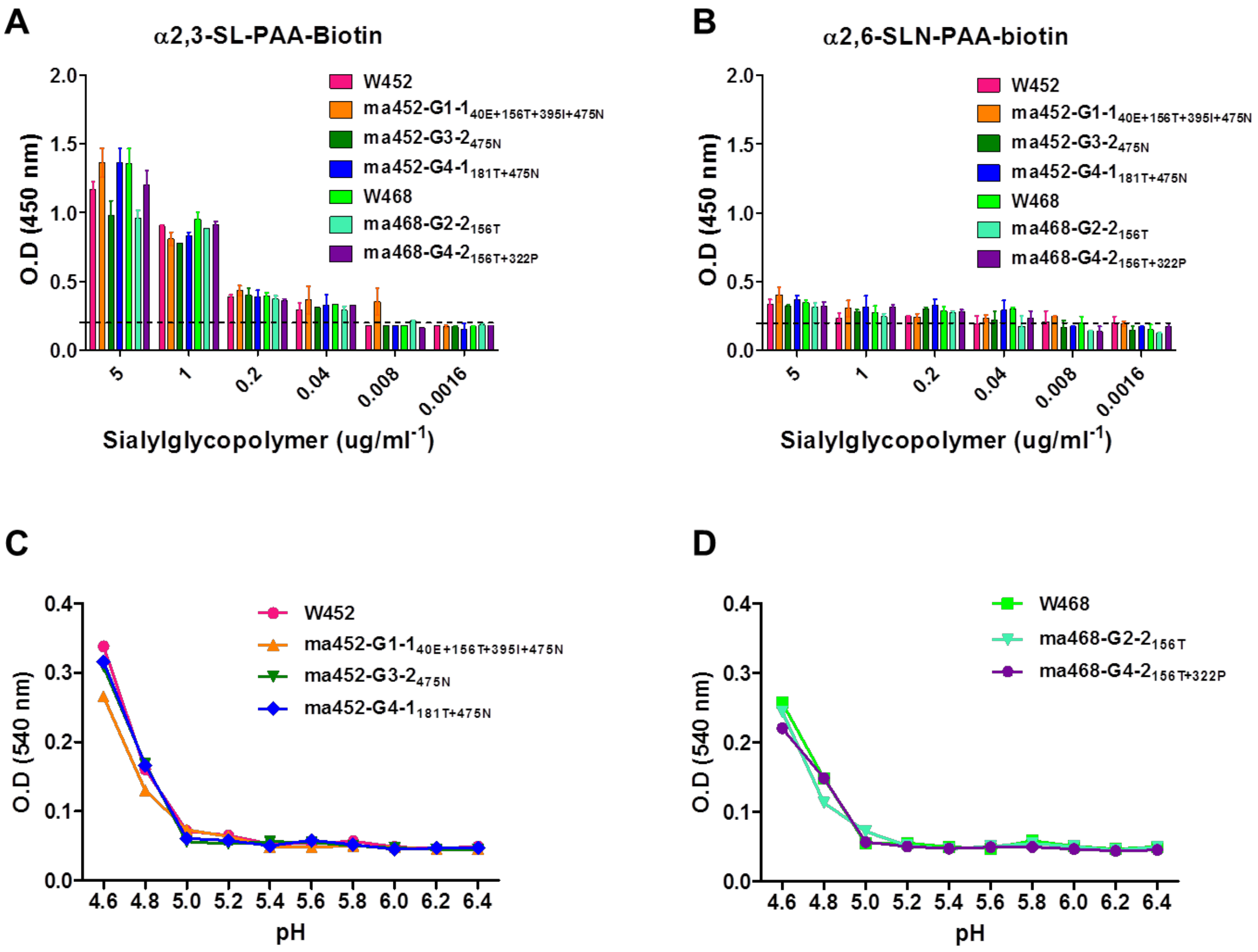

Supplementary Figure S8. Virus receptor-binding specificity and pH-dependent haemolysis assays for wild-type and mouse-adapted H5N8 mutants. Binding affinity of inactivated wild-type and mouse-adapted 452 and 468 viruses to sialic acid (SA) α2,3′-SL-PAA-biotin (**A**), and SA α2,6′SLN-PAA-biotin (**B**) glycans. The results shown are means±SD. The limits of detection are indicated by the dashed lines. A pH-dependent haemolysis assay for wild-type and mouse-adapted 452 (**C**) and 468 (**D**) viruses was conducted in which the pH of the acidic buffer ranged between 4.6 and 6.4. The degree of haemolysis was expressed as the optical density at 540 nm (OD 540) minus the blank value.

Supplementary Tables

Supplementary Table S1. Amino acid sequence homology between W452 and W468 viruses.

Gene Segment	Genetic identity (genetic distance)
PB2	99.6 (0.3)[a]
PB1	99.5 (0.4)
PA	99.7 (0.3)
HA	98.9 (0.9)
NP	99.4 (0.6)
NA	99.4 (0.4)
M	99.6 (0.0)
NS	98.9 (0.8)

[a] Genetic identities (genetic distance)

Abbreviations: HA, haemagglutinin; M, matrix; NA, neuraminidase; NP, nucleoprotein; NS, nonstructural; PA, polymerase; PB1, polybasic 1; PB2, polybasic 2

Supplementary Table S2. Amino acid sequence comparison between wild-type and mouse-adapted H5N8 viruses after 5 passages.

Virus	PB2				PB1	PA				HA[a]						NP	
	4	591	627	701	708	4	86	97	556	40	156	181	322	395	475	448	485
W452	I	Q	E	D	P	F	M	T	Q	K	A	S	L	V	K	M	G
ma452 G1	M	K	-	N	S	-	I	I	-	E	T	-	-	I	N	-	-
ma452 G2	-	-	-	N	S	V	-	-	-	-	-	-	-	-	N	I	-
ma452 G3	-	-	-	N	-	-	-	-	R	-	-	-	-	-	N	-	-
ma452 G4	-	-	-	N	-	-	-	-	R	-	-	T	-	-	N	-	-
W468	I	Q	E	D	P	F	M	T	Q	K	A	S	L	V	N	M	G
ma468 G1	-	-	K	-	-	-	-	I	-	-	-	-	-	-	-	-	R
ma468 G2	-	-	K	-	S	-	-	-	-	-	-	-	-	-	-	-	-
ma468 G3	-	-	K	-	S	-	-	-	-	-	T	-	-	-	-	-	-
ma468 G4	-	-	K	-	S	-	-	-	-	-	T	-	P	-	-	-	-

[a]H3 numbering

Abbreviations: A, alanine; D, aspartic acid; E, glutamic acid; F, phenylalanine; G, glycine; HA, haemagglutinin gene; I, isoleucine; K, lysine; L, leucine; M, methionine; ma, mouse-adapted; N, asparagine; P, proline; PA, polymerase acidic; PB1, polymerase basic 1; PB2, polymerase basic 2; Q, glutamine; R, arginine; S, serine; SD, standard deviation; T, threonine; V, valine

Supplementary Table S3. Amino acid polymorphism of mutations identified in mouse-adapted H5N8 viruses.

Protein	Amino acid change[a]	Clones(s)	Polymorphism(s) in database (frequency)[b]					
			HPAI H5Nx[c]	# Sequences	Avian	# Sequences	Human	# Sequences
PB2	I4M	ma452 G1	I (100)	333	I (98.086), E (0.512), Q (0.463), K (0.187), G (0.147), N (0.049), V (0.041), D (0.033), T (0.025), M (0.017), R (0.009)	12325	I (99.873), D (0.064), L (0.019), N (0.01), V (0.01)	10976
	Q591K	ma452 G1	Q (99.7), K (0.301)	333	Q (98.26), V (0.211), K (0.175), L (0.606), T (0.321), H (0.219), R (0.146), P (0.043), E (0.014)	13687	Q (58.61), R (41.10), S (0.143), K (0.075), L (0.037), H (0.015), F (0.007)	13245
	E627K	ma468 G1 ma468 G2 ma468 G3 ma468 G4	E (99.4), K (0.601)	333	E (94.82), K (3.795), T (0.210), V (0.916), L (0.130), R (0.036), A (0.021), G (0.021), Q (0.014), M (0.014), D (0.007), I (0.007)	13752	K (56.88), E (42.87), Q (0.143), R (0.082), A (0.015)	13265
	D701N	ma452 G1 ma452 G2 ma452 G3 ma452 G4	D (99.7), N (0.301)	333	D (99.45), T (0.206), E (0.235), N (0.088), A (0.007), V (0.007)	13573	D (99.44), N, (0.387), Q (0.144), E (0.015), Y (0.007)	13178
PB1	P708S	ma452 G1 ma468 G2 ma468 G4	P (100)	350	P (99.98), S (0.007), A (0.007)	12886	P (99.97), S (0.023)	12890
PA	M86I	ma452 G1	M (99.692), V (0.309)	348	M (92.071), L (6.266), I (0.979), V (0.588)	13565	M (99.48), V (0.296), I (0.181), L (0.03), K (0.008)	13488
	T97I	ma452 G2 ma468 G1	T (100)	348	T (99.27), N (0.281), A (0.190), I (0.137), S (0.106), P (0.007)	13122	T (99.69), S (0.085), A (0.085), N, (0.065), I (0.058), P (0.006)	15283
	Q556R	ma452 G3 ma452 G4	Q (100)	348	Q (99.49), H (0.225), L (0.091), R (0.063), N (0.035), P (0.028), M (0.007), K (0.007), D (0.007)	14198	Q (99.77), H (0.059), R (0.051), P (0.029), K (0.007)	13494
HA[d]	K40E	ma452 G1	K (80.61), R (19.391)	361	K (95.581), R (4.284), N (0.068), I (0.017), S (0.017), T (0.017)	5906	K (98.718), R (0.428)	468

	A156T	ma468 G4	A (95.845), T (4.156)		A (58.52), T (35.11), V (4.612), S (1.028), E (0.497), I (0.085), M (0.068), K (0.051), P (0.017)	5832	T (65.45), A (33.04), S (1.502)	466
	S181T	ma452 G1<br>ma468 G3<br>ma468 G4	S (95.291), P (4.71)	361	P (83.394), S (16.361), T (0.099), A (0.017), F (0.017), R (0.017)	6088	P (85.654), S (14.133)	467
	L322P	ma468 G4	L (91.136), Q (8.034), P (0.832)	361	Q (85.90), L (13.39), N, (0.150), K (0.150), P (0.083), V (0.050), I (0.016), S (0.016), E (0.016)	5973	Q (85.50), L (13.64), H (0.426), S (0.213), R (0.213)	469
	V395I	ma452 G1	V (99.169), I (0.832)	361	V (62.114), A (35.693), I (1.079), T (0.659), Y (0.037), S (0.22), D (0.019)	5469	V (99.571) I (0.43)	466
	K475N	ma452 G1<br>ma452 G2<br>ma452 G3<br>ma452 G4	N (99.723), K (0.278)	361	N (94.402), D (5.431), K (0.075), Q (0.019), T (0.019), Y (0.019)	5359	N (97.593), D (2.408), K (0.219)	457
**NP**	M448I	ma452 G2	M (100)	340	M (99.93), R (0.031), L (0.015), I (0.007), S (0.007)	12844	M (100)	13586
	G485R	ma468 G1	G (100)	340	G (99.977), E (0.012), R (0.012)	12833	G (99.932), R (0.029), D (0.023), E (0.012), S (0.006)	13546

[a]Amino acid substitutions found during mouse adaptation of H5N8 viruses

[b]Single-nucleotide polymorphisms of substituted positions in viral proteins; the ratio represents the prevalence of the amino acid to all relevant sequences in the Influenza Research Database.

[c]Prevalence of the amino acid in all highly pathogenic avian influenza H5Nx sequences, except HPAI H5N1.

[d]H5HA sequences were compared in H5Nx, avian and human H5 isolates.

Supplementary Methods

Chicken embryonic fibroblast cell culture. Chicken embryonic fibroblasts (CEFs) were derived from 10-day-old chicken embryos following a published protocol[2]. Briefly, a 10-day-old embryonic chicken was placed into a sterile petri dish and dissected using sterile scissors. After the contaminating blood/yolk was removed, the chopped embryo tissues were mixed with 25 mL of 0.25% trypsin and incubated for 20 min at 37°C with gentle shaking. Then, 1×10^6 CEFs were seeded into a 75-mL flask in Dulbecco's modified Eagle's medium (DMEM; Gibco-Invitrogen, Carlsbad, CA) containing 10% foetal calf serum (FCS; Omega) and 1% antibiotics (penicillin/streptomycin, Gibco-Invitrogen). After a 24-hour incubation, the supernatant was replaced with the growth medium, and the CEFs were maintained as previously described[2].

Minigenome assay for polymerase activity. A luciferase activity–based minigenome reporter assay was performed as described by Song et al.[3]. The human polymerase I promoter in a luciferase reporter plasmid (pHW72-Luci) was replaced with the chicken polymerase I promoter (cPolI), as previously described[4]. RNP complexes composed of pHWcPolI-Luc, pHW2000-PB2, pHW2000-PB1, pHW2000-PA, and pHW2000-NP, (0.1 μg each) were mixed with pCMV–β-galactosidase plasmids and then cotransfected into CEFs, which were prepared in 24-well plates 24 h prior to the transfection, using TransIT-LT1 transfection reagent as directed. After 6 h, the transfection medium was replaced with fresh, complete DMEM. The luciferase activity was measured at 48 h posttransfection. The cells were washed with PBS and lysed for 30 min with 100 μL of lysis buffer (Promega). Cell lysates were then harvested, and luciferase activity was assayed in triplicate using the luciferase assay system (Promega). The β-galactosidase activity was detected for each sample and used for normalization of minigenome expression between samples.

cDNA synthesis from total RNA and Real-time PCR. Total RNA was extracted from mice lungs infected with viruses using RNeasy mini kit (Qiagen) and cDNA was synthesized using the M-MLV Reverse Transcriptase kit (Enzynomics) following the manufacturer's suggested protocol, with random hexamer and Oligo-dT primers. Real-time PCR of IFN-alpha, IL-2, IL-6, and TNF-a genes was performed in 96 well optical plates (Sorenson Bioscience, Inc., Salt Lake City, UT, USA) with a master mix using SYBR green supermix according to the manufacturer's suggested protocol (Bio-Rad Laboratories). A GAPDH was included for normalization. Data analysis was performed using Bio-Rad CFX Manager™ Software.

Receptor-binding affinity. The HA-receptor affinity was determined in a solid-phase direct virus-binding assay, as previously described[5]. Briefly, influenza viruses were bound to fetuin-coated plates overnight at 4 °C. Biotinylated glycans (α2,3′SL; α2,6′SLN; Glycotech Corporation, Gaithersburg, MD) were added to influenza-coated plates via serial dilutions and were incubated for 4 h at 4 °C. Horseradish peroxidase–conjugated streptavidin (Invitrogen, Carlsbad, CA) was added, and 3,3′,5,5′-tetramethylbenzidine substrate (Sigma-Aldrich, St Louis, MO) was used as a substrate. Absorbance at 450 nm was measured in a VICTOR3 1420 multilabel counter plate reader (Perkin-Elmer, Waltham, MA).

Haemolysis assay. Standardized virus stocks (256 HA units in 50 μL) were diluted in 2% chicken red blood cells (RBCs) at a ratio of 1:4 and incubated on ice for 1 h to allow binding of virus to cells. Next, the RBCs (with adsorbed virus) were centrifuged (72 ×*g* at 4 °C for 3 min). Pelleted cells were resuspended in 200 μL of pH-adjusted PBS buffer and incubated at 37 °C for 1 h to allow activation of the HA protein and fusion. Intact chicken RBCs were removed by centrifugation (72 ×*g* at 4 °C for

3 min,). Cells were pelleted, and the amount of fusion-induced cell lysis was determined as a function of haemoglobin released into the supernatant by measuring absorbance at 540 nm.

Reference List

1 Reed, L. J. & Muench, H. A simple method of estimating fifty percent endpoints. *Am J Hyg* **27**, 493-497 (1938).

2 Hernandez, R. & Brown, D. T. Growth and maintenance of chick embryo fibroblasts (CEF). *Curr.Protoc.Microbiol.* **Appendix 4**, 4I (2010).

3 Song, M. S. *et al.* The polymerase acidic protein gene of influenza a virus contributes to pathogenicity in a mouse model. *J.Virol.* **83**, 12325-12335 (2009).

4 Massin, P., Rodrigues, P., Marasescu, M., van der Werf, S. & Naffakh, N. Cloning of the chicken RNA polymerase I promoter and use for reverse genetics of influenza A viruses in avian cells. *J.Virol.* **79**, 13811-13816 (2005).

5 Kim, Y. I. *et al.* Pathobiological features of a novel, highly pathogenic avian influenza A(H5N8) virus. *Emerg.Microbes.Infect.* **3**, e75 (2014).
